# Dimensional stability of pet-g clear aligners with different thickness through cyclic compressive load tests: An *in vitro* study

**DOI:** 10.4317/jced.62842

**Published:** 2025-07-01

**Authors:** Mauro Lorusso, Michele Tepedino, Gianluca Rendina, Angela Pia Cazzolla, Fariba Esperouz, Lucio Lo Russo, Domenico Ciavarella

**Affiliations:** 1 Department of Clinical and Experimental Medicine, Dental School of Foggia, University of Foggia, Foggia, Italy; 2 Department of Biotechnological and Applied Clinical Sciences, Dental School of L’Aquila, University of L’Aquila, L’Aquila, Italy

## Abstract

**Background:**

The purpose of this *in vitro* study was to evaluate the dimensional stability of PET-G clear aligners.

**Material and Methods:**

Two starting passive aligners of different thicknesses (0.75 mm and 0.88 mm) were subjected to cyclic compressive loading tests using the Instron 3343 testing machine in the laboratory, both with and without saliva. The intermolar and intercanine distances, measured in the occlusal direction, and the intermolar and intercanine Facial Axis (FA) distances, calculated between the most vestibular points of the crowns defined as Facial Axis points, were recorded using the dedicated software 3Shape Orthoviewer. Finally, the aligners were scanned using a laboratory scanner (3Shape TRIOS®) and then compared with the untested aligners of the control group.

**Results:**

The 0.75-mm-thick aligners exhibited greater dimensional stability than the 0.88-mm-thick aligners when measuring intercanine distance, both with and without saliva. In contrast, the 0.88-mm-thick aligners demonstrated greater stability than the 0.75-mm-thick aligners when measuring intermolar distance under the same conditions. In the measurement of FA distances, higher values were observed at the molar level when the tests were conducted both with and without saliva, whereas no significant changes were observed in the intercanine distance.

**Conclusions:**

The 0.88-mm-thick aligners demonstrated better dimensional stability during tests with and without saliva in the intermolar distance, while the 0.75-mm-thick aligners showed better dimensional stability in the intercanine distance.

** Key words:**Clear Aligners, PET-G, thickness, dimensional stability, cyclic compressive load tests.

## Introduction

Orthodontic treatments are increasingly influenced by esthetic considerations (1), which, in combination with social perception, have led to the development of new methods for treating malocclusions as an alternative to traditional fixed braces, such as esthetic brackets, lingual appliance, and clear aligners (2). A growing number of adolescent and adult patients require aesthetic treatments that are compatible with their daily lives and do not affect their quality of life (3). The use of functional devices to correct malocclusions is decreasing (4,5), while the use of clear aligners and auxiliaries to address sagittal discrepancies is becoming more prevalent. In particular, clear aligners are widely used because they facilitate orthodontic movements through controlled force distribution, gradually realigning teeth according to a specific orthodontic treatment plan (6). However, during orthodontic treatment, it is important to respect the arch form, particularly avoiding changes to the intercanine distance to prevent relapse, and to carefully analyze occlusal forces, which in some cases can hinder tooth movement (7,8).

The conventional process of making an aligner is based on obtaining a model of a patient’s dental impression, followed by subsequent thermoforming. Inaccuracies are common and are often caused by errors in the impression-taking and thermoforming processes. These inaccuracies could be minimized through the use of digital technologies and printed devices that do not require thermoforming.

Recently, the direct printing of clear aligners has been introduced to improve manufacturing accuracy and reduce errors. One of the significant advantages of directly printed aligners is their design flexibility, including the ability to adjust material thickness and control the offset between the aligner and the teeth (9).

Clear aligners are made of thermoplastic materials, whose composition and biomechanical properties are key factors affecting their clinical performance in the oral environment (10). The most commonly used thermoplastic material is glycol-modified polyethylene terephthalate (PET-G), an amorphous polyester with high impact resistance, chemical resistance, and flexural strength. PET-G is produced by adding a cyclohexane dimethanol molecule to the PET backbone instead of ethylene glycol, resulting in a copolymer with a lower melting temperature. This modification enhances strength, durability, and impact resistance, making it more suiTable for high-temperature applications (11).

Due to its low forming temperature, PET-G can be easily vacuum-formed, pressure-formed, or heat-bent. These properties make it one of the most widely used materials for 3D printing and other heat-forming processes. In the dental field, PET-G is particularly valued for the fabrication of orthodontic devices and occlusal appliances (12) . However, other materials such as polypropylene (PP), polycarbonate (PC), thermoplastic polyurethanes (TPU), and ethylene vinyl acetate (EVA) can also be used. Biomechanical properties include elasticity, low hardness, resilience, dimensional stability, transparency, low cytotoxicity, high biocompatibility, and resistance to the effects of saliva and oral temperature (13). To date, many thermoplastic polymers are available on the market, but they have different mechanical and chemical characteristics (14) . During the wear period, various factors within the oral cavity can alter material properties over time, such as the action of salivary enzymes, humidity and temperature changes, and continuous or intermittent forces caused by normal oral functions such as chewing, speaking, swallowing, and parafunctional activities (15).

Another key factor that can influence the mechanical properties and, consequently, the clinical performance of the material is the thickness. Currently, aligners are available in a range of customizable thicknesses, from 0.50 mm to 1.5 mm, tailored to treatment needs. Aligners made from thicker materials (>0.75 mm) generate significantly higher forces compared to those made from thinner materials (<0.5 mm) (16). An increase in thickness results in greater force delivery while simultaneously reducing the flexural modulus. As a result, the performance of clear aligners can vary depending on both their thickness and the materials used in their construction. In an ideal situation, an aligner should consistently apply a light force overtime (17). To exert safe and effective forces, the ideal material should be relatively stiff with a high yield strength, ensuring that the force applied remains within the elastic range. The stress relaxation curve should, therefore, be reasonably flat, demonstrating the material’s ability to exert constant and continuous forces over time.

Since the characteristics required for a thermoplastic material to be used in the orthodontic field are numerous and cannot be found in a single material (18), there is currently no standard method for evaluating all the mechanical and chemical properties of these materials. For this reason, the aim of this study was to evaluate variations in the dimensional stability of PET-G clear aligners with different thicknesses through in-vitro studies based on cyclic compressive load stress, conducted both in the presence and absence of saliva.

## Material and Methods

A 27-year-old male patient, treated at the Department of Orthodontics at the University of Foggia, was selected and enrolled in this study after signing informed consent, considering the following criteria: presence of all teeth and good periodontal health. The exclusion criteria were as follows: agenesis, periodontal disease, fixed or removable prostheses, loss of one or more teeth, oral parafunctions and signs or symptoms of temporomandibular joint disorders. The patient selected for this study exhibited moderate incisor crowding in both arches and presented good oral hygiene.

A direct scan of the maxillary and mandibular arches was performed using an intraoral scanner (TRIOS; 3Shape, Copenhagen, Denmark), following the manufacturer’s recommended protocol. The impression models were used to thermoform two PET-G aligners with thicknesses of 0.75 mm and 0.88 mm. Three copies of the first aligner in each series, programmed as passive aligners, were used for tests performed with saliva, without saliva, and in a control group, respectively.

- Testing protocol

**Figure 1:**
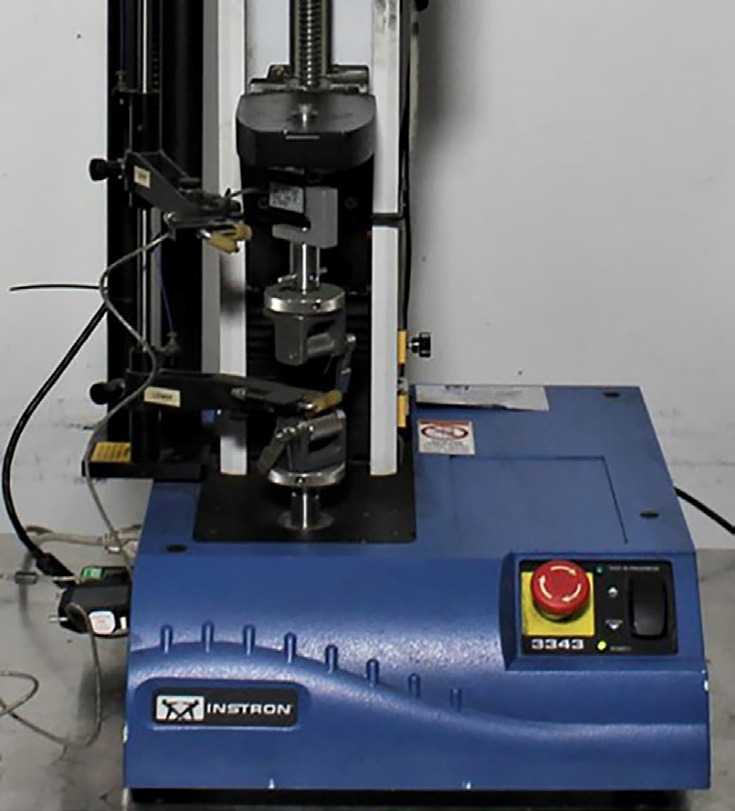
Instron 3343 Universal Testing Machine.

For testing, the models were placed in occlusion and then secured with clamps onto the two metal plates of the Instron 3343 universal testing machine (Fig. 1) with a 1 kN load cell (ISO). The Instron testing machine features electromechanical and hydraulic systems for performing static tests planned by the Bluehill software, including tensile, compression, bending, peeling, tearing, shearing, friction, puncture, and other mechanical tests.

The tests were performed both in the presence of artificial saliva (prepared as a galenic formulation in a pharmacy) using a soaked sponge placed between the upper and lower study models and secured around the perimeter of the two metal plates with films, and without saliva.

**Figure 2:**
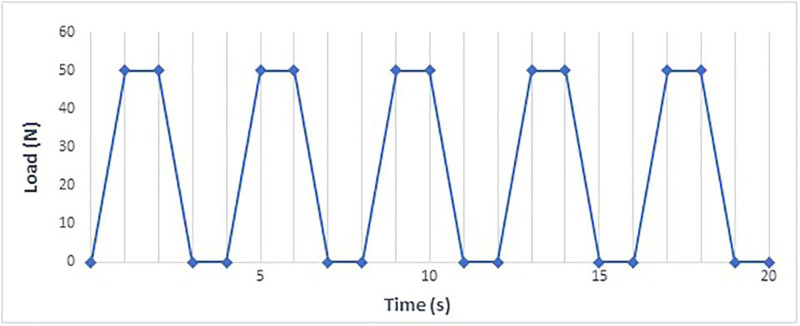
Graphic representation of the load cycles.

These cyclic compression tests, conducted at room temperature (25°C) in the research laboratory of the Polytechnic of Bari, consisted of four phases, as listed below (Fig. 2):

1. Starting from zero (0 N), the compressive load reaches 50 N in one second, a value corresponding to the occlusal forces generated by the opening and closing movements of the human jaw during swallowing;

2. Hold the load for one second to ensure maximum occlusal contact;

3. Reduce the load back to zero (0 N) within one second;

4. Maintain the load at zero (0 N) for one second.

Each aligner was subjected to 22,.500 cycles to simulate the number of occlusal contacts during swallowing over two weeks of aligner use. These cycles were divided over four days, averaging approximately 6,000 cycles per day, following sensor calibration.

**Figure 3:**
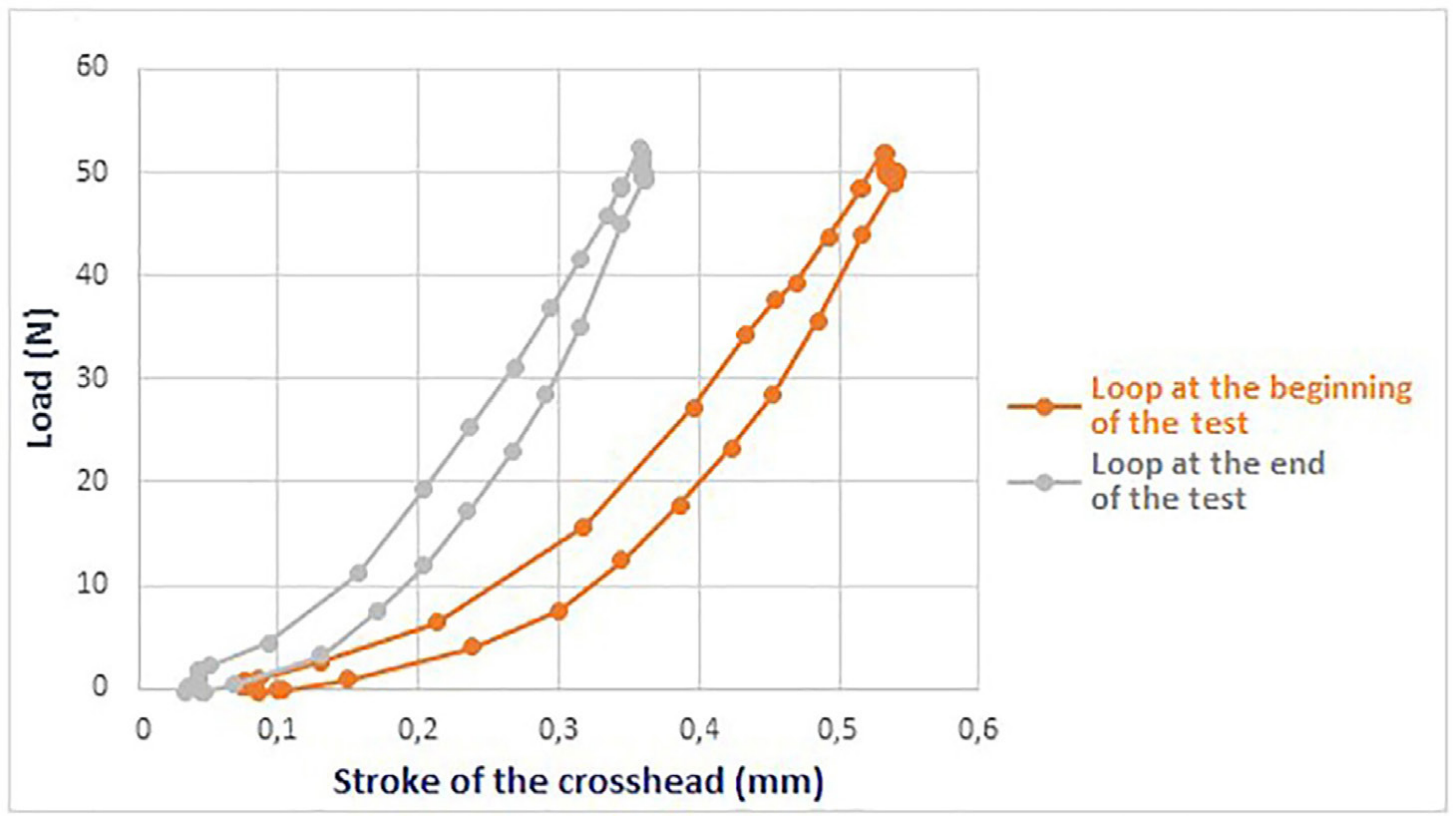
Graph illustrating the gradual decrease in the crosshead’s stroke throughout the test.

The Instron 3343 single-column testing machine uses a rotating ball screw to drive a load-bearing crosshead up and down. An electric motor powers a series of pulleys and gears that turn the screw, generating the crosshead motion. The compression tests were performed under load control, ensuring that “the stroke of the crosshead”, the maximum distance between the two metal plates at the end of each compression cycle, was not predetermined. The machine achieved the specified load of 50 N within the designated time frame. For this reason, the stroke of the crosshead did not remain constant; instead, a reduction was observed during the tests, likely due to the material’s stiffening (Fig. 3).

Figure 3 shows that at the beginning of the test, the crosshead stroke required to reach the preset load of 50 N is higher (0.53 mm), whereas by the end of the test, the required stroke decreases to 0.36 mm. The tested aligners did not exhibit evident tear points or perforations, as the load was distributed over a wider surface, thereby reducing the localized pressure at the contact points.

- Data collection

At the conclusion of the tests, both the tested aligners and those in the control group were first coated with Helling 3D Spray for scanning. They were then scanned using a laboratory scanner (3Shape TRIOS® ScanIt Manager TPS Ink, 3Shape, Copenhagen, Denmark) and subsequently analyzed with specialized software (3Shape OrthoViewer, 3Shape, Copenhagen, Denmark) for the digital detection of linear measurements, specifically the distance between the canines (anterior region) and between the molars (posterior region).

The intercanine distance, measured between the tips of the contralateral canines, and the “intermolar distance,” calculated between the occlusal grooves of the contralateral molars, were first measured to assess the deformation of the thermoplastic devices in the occlusal sense. Finally, the intercanine and intermolar distances, measured between the most vestibular points (Facial Axis points, FA) on the canine and molar crowns, were recorded to evaluate changes in the inclination of the dental arches.

## Results

**Figure 4:**
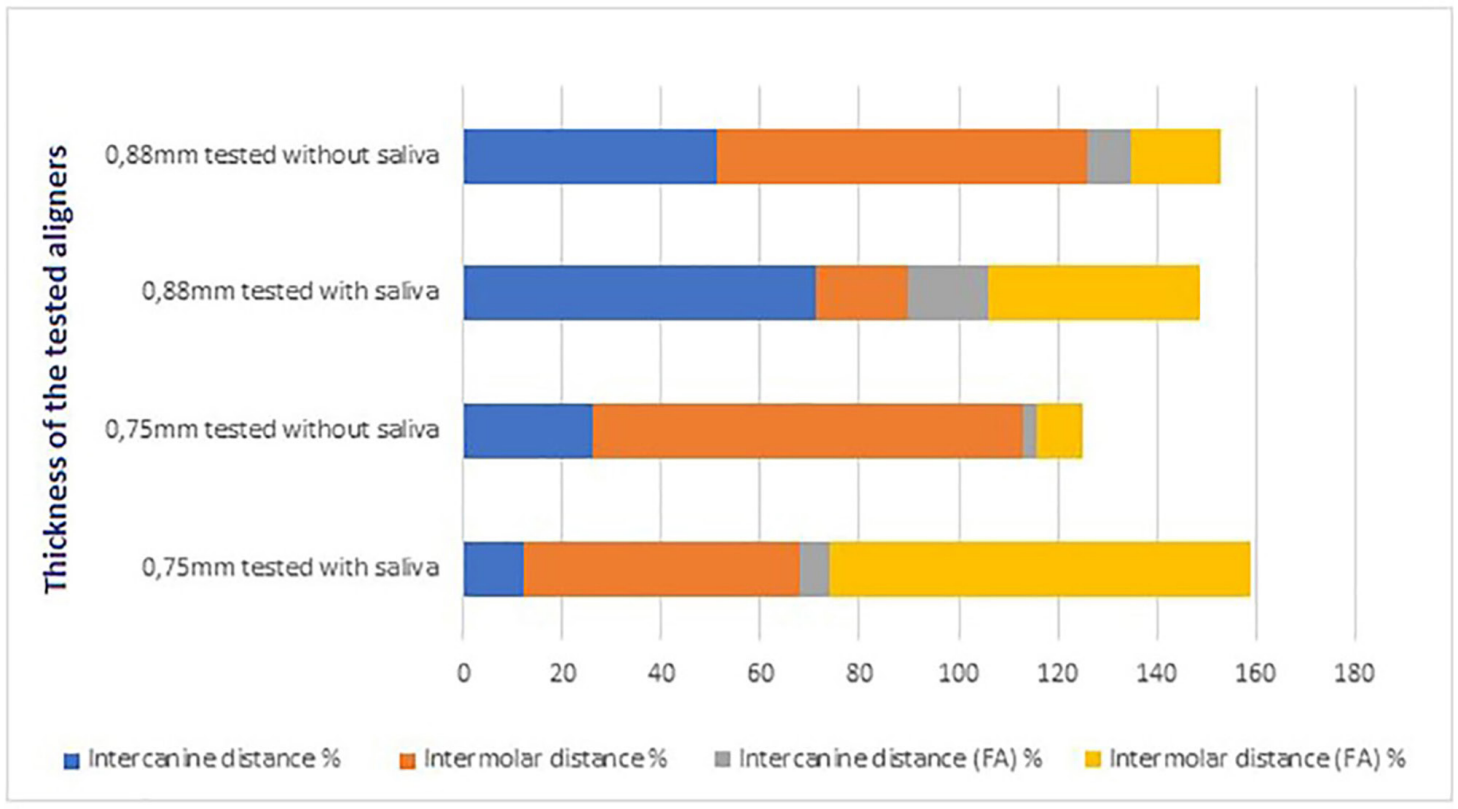
Total deformation obtained by summing the variations of the four distances measured for each aligner in both test conditions.

The measurement of both types of intercanine and intermolar distances of each study group were reported in the  Table 1 and  Table 2. Tables  Table 3 and  Table 4 show the differences in quantitative deformation values between the aligners tested both in presence and in absence of saliva compared to the untested ones. The percentage differences in deformation of the aligners with a thickness of 0.75 mm and 0.88 mm, both with and without saliva, in the control group are shown in  Table 5 and  Table 6. Finally, Figure 4 shows the histogram of the overall deformation values, which represent the sum of all measured distances.

In the intercanine distance, aligners with a thickness of 0.75 mm exhibited a higher strain value of 0.00014 µm when tested without saliva, compared to those tested with saliva. In the intermolar distance measurement, a higher strain value of 0.00031 µm was observed for aligners tested without saliva, compared to those tested with saliva.

In the aligners with a thickness of 0.88 mm, for the intercanine distance, a deformation value 0.00020 µm higher was found in those tested with saliva compared to those tested without saliva. Meanwhile, in the intermolar distance, a deformation value 0.00056 µm higher was observed for those tested without saliva compared to those tested with saliva.

Instead, in the intercanine FA distance for the aligners with a thickness of 0.75 mm, a higher deformation value of 0.00003 µm was found in the aligners tested in the presence of artificial saliva compared to those tested without saliva. Similarly, in the measurement of the intermolar FA distance, a 0.00096 µm higher deformation value was observed in the aligners tested with saliva compared to those tested without saliva.

For the intercanine FA distance for the aligners with a thickness of 0.88 mm, a 0.00016 µm higher deformation value was detected in the aligners tested in the presence of saliva compared to those tested without saliva. Meanwhile, in the measurement of the intermolar FA distance, a 0.00025 µm higher strain value was found in the aligners tested with saliva compared to those tested in the absence of saliva.

## Discussion

Understanding the potential and limitations of a therapeutic device based on its mechanical properties is crucial for the proper functioning of the therapy and, consequently, for obtaining predictable results. To be effective and prevent relapse, aligners must retain their structural integrity and physical properties throughout the wear period (19). Mechanical wear of clear aligners, which involves the loss of material from solid surfaces due to mechanical interaction, is a complex process in the clinical context because of individual patient patterns of occlusal interaction and parafunctional habits (20). These dynamics are more difficult to predict and control in the oral cavity than in a laboratory environment, where aligners are subjected to tests. For this reason, this paper aims to understand the mechanical behavior of PET-G clear aligners with different thicknesses through compression load tests performed both in the presence and absence of artificial saliva in a laboratory environment to evaluate their dimensional stability. The 0.75 mm thick aligners exhibited lower strain values in terms of dimensional stability on the vertical plane compared to the 0.88 mm thick aligners when measuring the intercanine distance, both with and without saliva. In contrast, the 0.88 mm thick aligners showed lower deformation values than the 0.75 mm thick aligners when measuring the intermolar distance in both testing conditions. Regarding the inclination of the dental arches, higher values were observed at the molar level in both the presence and absence of saliva, whereas no changes were observed in the canine region. These findings are likely due to the “bite-block effect” (21,22). Specifically, the bite-block effect temporarily causes the creation of an iatrogenic posterior open bite as a result of molar intrusion (23) , leading to a reduction in occlusal contacts and posterior occlusal surfaces (24) , due to the thickness of the two thermoplastic devices, which resulted in the formation of pre-contacts anteriorly. Horton *et al*. (25) found a reduction in occlusal contacts when using an essix occlusal-covered device compared to a Hawley retainer. Subsequently, after the end of the treatment, a “settling of the occlusion” occurs, with an increase in occlusal contacts and occlusal surfaces (26,27). For all the distances analyzed, it was observed that the 0.75 mm thick aligner generally exhibited the highest deformation values when the tests were performed in the presence of saliva, suggesting lower dimensional stability compared to the 0.88 mm thick aligner, which, in contrast, showed lower strain values both with and without saliva. Therefore, the aligner with a thickness of 0.75 mm has a shorter lifespan compared to the 0.88 mm one within the same clinical condition. Consequently, it is important to replace the aligner more frequently to avoid a reduction in its clinical performance. Kohda N. *et al*. (16) showed that the thickness of the materials had a significant influence on the forces delivered by the thermoplastic appliances for the tipping movement of the left central maxillary incisor when comparing three different thermoplastic materials with two different thicknesses, highlighting how thicker aligners provide greater forces.

Casavola *et al*. (28) analyzed the mechanical deformation of PET-G clear aligners under cyclic compression tests using Digital Image Correlation (DIC) and Optical Microscope (OM) analysis to study two PET-G aligners thermoformed with thicknesses of 0.75 mm and 0.88 mm, respectively. The aligners were subjected to cyclic compression up to 13.000 load cycles, ranging from 0 to 50 N at room temperature, in order to simulate the average load to which an aligner is subjected over one week. Both aligners showed greater displacements in the early stages of loading, with the 0.88 mm aligner showing more pronounced changes. The OM analyses revealed significant signs of wear and tear, including deep depressions and cracks on the aligners’ surfaces, especially for the 0.75 mm thickness.

Barile *et al*. (29) analyzed the performance of thermoplastic materials under different cycles of compressive loading using the Acoustic Emission (AE) technique, to identify the cycles at which the aligners may losing their functionality. Three different specimens of PET-G, PU (polyurethane), and additively manufactured PU underwent 22,500 cyclic compression tests to simulate swallowing mechanisms. The mechanical results showed that the energy absorbed by the thermoformed PET-G aligners remained stable at around 4 Nmm throughout the tests. Although a higher energy absorption of about 7 Nmm was observed for the PU-based aligners during the initial phase of the cyclic loading, this gradually decreased after 12,500 cycles. The mechanical test results indicate that thermoformed PET-G has a higher potential to meet the performance and requirements of thermoplastic materials in the orthodontic field.

Cianci *et al*. (30) evaluated the mechanical behavior of PET-G aligners with two different thicknesses, 0.75 mm and 0.88 mm subjected to cyclic compression tests in an atmospheric environment (25°C) as well as in the presence of saliva. The tests were performed using a testing machine, and each aligner was subjected to 22,500 load cycles ranging from 0 to 50 N. Specifically, an increase in the aligners’ stiffness and a gradual reduction in crosshead displacement were observed during the tests, highlighting the occurrence of cyclic hardening phenomena. It was also found that, within the analyzed range of load rates, the aligners exhibited residual strain recovery after the applied load was removed and showed a low tendency to accumulate residual strains as loading cycles progressed.

Jindal *et al*. (31) investigated the performance of various thermoplastic materials, both thermoformed and 3D printed, when subjected to mechanical compressive loading equivalent to human biting forces. Specifically, they compared the material properties of conventional dental aligner materials, such as Duran and Durasoft, with Formlabs Dental Long Term LT Clear resin. The authors concluded that 3D printed, resin-cured clear dental aligners are more suiTable due to their better geometric accuracy and mechanical resistance, although clinical data to evaluate the performance and durability of Dental LT resin during patient use are lacking in the literature.

Limitations of the study

Since the study was conducted in a laboratory, it did not account for the natural space of the periodontal ligament and its elastic behavior, which likely modulate the clinical performance of the aligner’s materials during the wearing period. Additionally, only passive starting aligners without attachments were included as specimens, meaning they were incapable of generating the orthodontic forces necessary for tooth movement. As a result, it was not possible to evaluate whether and how the dimensional stability of the subsequent aligners with different geometries, compared to the initial ones, changed in the later phases of treatment. Finally, since only thermoformed aligners made of PET-G were analyzed, no comparison was made with other thermoplastic materials or their combinations, which, due to their chemical and mechanical properties, could respond differently to stress. Further studies are necessary to fully understand how the dimensional stability of PET-G clear aligners changes as their thickness varies, particularly through *in vivo* studies under intraoral conditions.

## Conclusions

The dimensional stability of PET-G dental aligners with different thicknesses, simulating occlusal contacts during normal swallowing movements by presetting the compression load at 50 N was analysed in the present study. At the end of the tests, an increase in the intermolar distance compared to the intercanine distance was observed, regardless of the aligner thickness tested or the methods used for the tests. This result is probably due to the presence of anterior premature contacts caused by the thickness of the two thermoplastic devices placed in the interocclusal space, which is one of the limitations during the initial phases of clear aligner treatment. The thickness of the aligner is one of the most important mechanical properties due to its impact on maintaining the device’s dimensional stability during the wear period, as well as on the magnitude of the forces delivered. Irregularities in thickness can influence the fitting accuracy of the aligner. Overall, the lower dimensional stability of the 0.75 mm aligner compared to the 0.88 mm aligner highlights the relationship between thickness, hardening, and the flexural modulus of the thermoplastic materials used to make these devices. This underscores the need for more frequent replacement due to the probable loss of their ability to exert constant and continuous forces over time within the elastic range.

## Figures and Tables

**Table 1:** Intercanine and intermolar distances, as well as intercanine FA and intermolar FA measurements, for 0.75-mm-thick aligners tested in the presence of saliva, without saliva, and in the control group.

0.75 mm thickness	INTERCANINE DISTANCE (µm)	INTERMOLAR DISTANCE (µm)	INTERCANINE FA DISTANCE (µm)	INTERMOLAR FA DISTANCE (µm)
Control group	0.03441	0.05016	0.03747	0.05338
With saliva	0.03453	0.05072	0.03753	0.05423
Without saliva	0.03467	0.05103	0.03750	0.05329

**Table 2:** Intercanine and intermolar distances, as well as intercanine FA and intermolar FA measurements, for 0.75-mm-thick aligners tested in the presence of saliva, without saliva, and in the control group.

0.88 mm thickness	INTERCANINE DISTANCE (µm)	INTERMOLAR DISTANCE (µm)	INTERCANINE FA DISTANCE (µm)	INTERMOLAR FA DISTANCE (µm)
Control group	0.03425	0.05072	0.03756	0.05348
With saliva	0.03496	0.05091	0.03772	0.05391
Without saliva	0.03476	0.05147	0.03756	0.05366

**Table 3:** Difference in measurement of 0.75-mm-thick aligners tested with and without saliva, compared to the control group.

0.75 mm thickness	INTERCANINE DISTANCE (µm)	INTERMOLAR DISTANCE (µm)	INTERCANINE FA DISTANCE (µm)	INTERMOLAR FA DISTANCE (µm)
With saliva- Control group	0.00012	0.00056	0.00006	0.00085
Without saliva-Control group	0.00026	0.00087	0.00003	-0.00009

**Table 4:** Difference in measurement of 0.88-mm-thick aligners tested with and without saliva, compared to the control group.

0.88 mm thickness	INTERCANINE DISTANCE (µm)	INTERMOLAR DISTANCE (µm)	INTERCANINE FA DISTANCE (µm)	INTERMOLAR FA DISTANCE (µm)
With saliva- Control group	0.00071	0.00019	0.00016	0.00043
Without saliva-Control group	0.00051	0.00075	0	0.00018

**Table 5:** Difference in measurement of 0.75-mm-thick aligners tested with and without saliva, compared to the control group, expressed as a percentage.

0.75 mm thickness	INTERCANINE DISTANCE (%)	INTERMOLAR DISTANCE (%)	INTERCANINE FA DISTANCE (%)	INTERMOLAR FA DISTANCE (%)
With saliva- Control group	12	56	6	85
Without saliva-Control group	26	87	3	9

**Table 6:** Difference in measurement of 0.88-mm-thick aligners tested with and without saliva, compared to the control group, expressed as a percentage.

0.88 mm thickness	INTERCANINE DISTANCE (%)	INTERMOLAR DISTANCE (%)	INTERCANINE FA DISTANCE (%)	INTERMOLAR FA DISTANCE (%)
With saliva- Control group	71	19	16	43
Without saliva-Control group	51	75	0	18

## Data Availability

The datasets used and/or analyzed during the current study are available from the corresponding author.
